# Myocardial Ischemia Assessment in Chronic Kidney Disease: Challenges and Pitfalls

**DOI:** 10.3389/fcvm.2014.00013

**Published:** 2014-12-19

**Authors:** Susie F. C. Parnham, Jonathan M. Gleadle, Carmine G. De Pasquale, Joseph B. Selvanayagam

**Affiliations:** ^1^Department of Cardiovascular Medicine, Flinders Medical Centre, Bedford Park, SA, Australia; ^2^School of Medicine, Flinders University, Bedford Park, SA, Australia; ^3^Department of Renal Medicine, School of Medicine, Flinders University, Bedford Park, SA, Australia

**Keywords:** chronic kidney disease, myocardial ischemia, coronary disease, echocardiography, myocardial perfusion imaging

## Abstract

Coronary artery disease is the leading cause of mortality and morbidity in the chronic kidney disease (CKD) population and often presents with atypical symptoms. Current diagnostic investigations of myocardial ischemia in CKD lack sensitivity and specificity or may have adverse effects. We present a case vignette and explore the challenges of diagnostic myocardial stress investigation in patients with CKD.

## Case Vignette

A 60-year-old woman with stage 5 chronic kidney disease (CKD) from glomerulonephritis presented with atypical chest discomfort at rest. She is worried about coronary artery disease (CAD). She has hypertension treated with an angiotensin converting enzyme inhibitor and a beta blocker. Her total cholesterol is 5 mmol/L with low-density lipoprotein of 3.2 mmol/L. She has no diabetes mellitus, no family history of premature CAD, and does not smoke. How should she be evaluated?

## Clinical Problem

Cardiovascular disease (CVD) is the leading cause of mortality and a major cause of morbidity in the CKD population. Myocardial ischemia is a major cause of death in CKD patients. Myocardial ischemia can be caused by both epicardial and microvascular CAD.

Coronary artery disease is highly prevalent in the CKD population (1) being evident even in early renal disease (2, 3) and in young CKD patients (4). CKD patients have both traditional and non-traditional cardiac risk factors (Table 1).

**Table 1:** **Cardiovascular risk factors in the chronic kidney disease population**.

Traditional cardiovascular risk factors	Non-traditional cardiovascular risk factors
Hypertension	Left ventricular hypertrophy
Diabetes mellitus	Fluid overload
Dyslipidemia	Uremia
Smoking	Anemia
Family history of coronary artery disease	Disorders of vitamin D, calcium, and phosphate
Age	Hyperparathyroidism
	Inflammatory state
	Proteinuria
	Nephrotic state

In the CKD population, CAD is often multi-vessel and causes silent or asymptomatic myocardial ischemia (5, 6). Asymptomatic epicardial CAD has been detected even in people with early stage CKD (7) and is associated with a higher major adverse cardiac event rate compared to those without CAD (6). While the coronary plaque characteristics in patients with CKD showed no difference in the prevalence of high-risk plaque compared to the group without CKD (8), Kawai et al. showed that patients with mild CKD had higher prevalence of severe epicardial CAD compared to those without CKD (8), thus, suggesting that the problem relates to coronary stenosis rather than plaque stability.

Microvascular CAD is also present in the CKD population. Charytan et al. assessed mild to moderate CKD subjects without diabetes or uncontrolled hypertension using positron emission tomography imaging and found that the CKD cohort had decreased coronary flow reserve (CFR) compared to controls (9). Chade et al. suggested that microvascular dysfunction occurred in early CKD (10). They performed coronary flow wire to the left anterior descending artery using adenosine on early CKD subjects with no angiographically significant CAD and found the CKD subjects had lower CFR compared to normal controls (10). Microvascular CAD has been shown to be associated with reduced survival, although, similar to patients without CKD, the rate of survival is better than for epicardial CAD (11).

## Strategies and Evidence

### Diagnostic evaluation

Diagnostic evaluation starts with a thorough clinical history and examination and a baseline 12-lead ECG. Cardiovascular examination, especially to exclude uncontrolled hypertension or significant aortic stenosis is important prior to cardiac stress investigation. Cardiac stress investigations in CKD patients and their limitations are outlined in Table 2.

**Table 2:** **Cardiac stress investigations in the normal renal function versus advanced chronic kidney disease (CKD) patients**.

Cardiac stress modalities	Sensitivity (%)		Specificity (%)		Issues
	Normal renal function	CKD	Normal renal function	CKD	
Exercise stress ECG	68 (52–84)	36 (21–54)	77 (60–94)	91 (83–96)	Reduced exercise capacity (deconditioning)
					Impaired chronotropic response
					Abnormal baseline ECG and left ventricular hypertrophy
Exercise stress echocardiography	71–97	Possibly similar to DSE	64–90	Possibly similar to DSE	Reduced exercise capacity (deconditioning)
					Impaired chronotropic response
					Abnormal baseline ECG and left ventricular hypertrophy
Pharmacological stress echocardiography	86 (78–91)	80 (64–90)	86 (75–89)	89 (79–94)	Blunted chronotropic response
					Left ventricular hypertrophy
					Microvascular disease potentially can be missed
Myocardial perfusion scintigraphy	89	69 (48–85)	75	77 (59–89)	False negative results in multi-vessel disease due to balanced ischemia
Dobutamine stress CMR		Research ongoing		Research ongoing	Blunted chronotropic response
					Microvascular disease potentially can be missed

### Exercise stress ECG

In patients with normal renal function, exercise stress test (EST) with ECG has a low to moderate sensitivity and specificity, 68 ± 16% and 77 ± 17%, respectively, even when adequate exercise capacity and 85% heart rate is achieved (12). EST is further limited in the advanced CKD population, with poor sensitivity of 36% (13) (especially those undergoing dialysis), as deconditioning leads to reduced exercise capacity (14). Deconditioning can be due to vascular, neurological or musculoskeletal comorbidities, and the catabolic/cachexic metabolic state associated with CKD. CKD patients have also been shown to have impaired heart rate response to exercise (15), and the frequently abnormal baseline ECG in CKD patients (often secondary to hypertension) hampers the interpretation of standard stress testing. In advanced CKD patients, the ST segment changes at stress were shown to be not significantly different between non-severe CAD and severe CAD group, despite a longer treadmill exercise time in the non-severe group (13).

### Exercise stress echocardiography and dobutamine stress echocardiography

Exercise stress echocardiography (ESE) is better than the standard stress ECG in ruling in CAD (Positive likelihood ratio ESE 7.94 versus EST 3.57) and ruling out CAD (Negative likelihood ratio ESE 0.19 versus EST 0.38) (16). Its sensitivity has been reported ranging from 71 to 97% with specificity ranging from 64 to 90% (17). However, the utility of ESE in CKD population remains limited due to the same physical reasons as EST limitations above.

The addition of echocardiography allows assessment of ventricular size and function, aortic and mitral valvular calcification, left ventricular hypertrophy (LVH), and potentially CFR. CFR measurement by Doppler echocardiography in the left anterior descending artery has been shown to be a determinant of cardiac events in CKD patients in the absence of obstructive epicardial CAD (18), although this is not performed routinely by many echocardiography laboratories due to technical difficulties.

Dobutamine and dipyridamole stress echocardiography (DSE) technique detects inducible myocardial ischemia based on detection of wall motion abnormalities, thus, would detect significant epicardial CAD, not microvascular disease.

A meta analysis in 2008 showed that dipyridamole and dobutamine stress echocardiography had a sensitivity of 85% (confidence interval 80–89) and 86% (confidence interval 78–91), respectively, and a specificity of 89% (confidence interval 82–94) and 86% (confidence interval 75–89), respectively, in detecting myocardial ischemia in the non-renal population (19). It is often recommended as a screening test in advanced CKD patients.

A systematic review in 2011 identified 11 DSE studies with 690 potential renal transplant recipients (19). Overall, DSE had moderate sensitivity of 80% (confidence interval 64–90) in detecting inducible myocardial ischemia in renal transplant candidates (20). Several mechanisms may explain the reduced accuracy of DSE in the advanced CKD population. The majority of advanced CKD patients had a blunted chronotropic response, thus, did not achieve 85% maximal predicted heart rate despite the use of atropine, significantly reducing the sensitivity of DSE in detecting myocardial ischemia (21). The thick myocardium due to LVH with small intracavitary volume, commonly found in CKD patients, obscures the detection of wall motion abnormalities at stress, thus, significantly reducing the sensitivity of stress echocardiography in detecting inducible myocardial ischemia in CKD population. Microvascular CAD is difficult to appreciate given the focus on regional wall motion abnormality and likely to be missed.

Abnormal DSE results in CKD patients have been associated with poorer prognosis for cardiac events and overall mortality (13, 22–24). Bergeron et al. showed that among 485 patients with CKD, the percentage of ischemic segments during DSE was an independent predictor of mortality (22). Negative stress echocardiography results, on the other hand, have been shown to be associated with low incidence of major adverse cardiac events (21).

Blunted chronotropic response with exercise in CKD population may relate to poorer overall cardiac prognosis. A 2012 meta analysis of 11,542 patients showed that submaximal age-predicted heart rate (<85% maximum heart rate) in the setting of normal ESE and DSE had higher cardiovascular risk than those who achieved >85% maximal predicted heart rate (25).

### Myocardial perfusion scintigraphy

Exercise and pharmacological myocardial perfusion scintigraphy (MPS) have sensitivity of 87 and 89%, and specificity of 73 and 75%, respectively, in detecting >50% coronary artery stenosis in patients without advanced CKD (26). Exercise MPS in the advanced CKD population has the same limitation as EST and ESE, i.e., related to the inadequate exercise performance and chronotropic incompetence (27).

A systematic review in 2011 showed that MPS has sensitivity of 69% (confidence interval 48–85) and specificity of 77% (confidence interval 59–89) in diagnosing inducible myocardial ischemia in the pre-renal transplant population (20). MPS has high false negative result in detecting ischemia in people with significant triple vessel CAD, as in the CKD population, because of homogeneous tracer uptake due to “balanced ischemia” (28, 29).

Normal myocardial perfusion measured by SPECT may not be associated with excellent prognosis in CKD population unlike the normal population (30, 31), perhaps due to the high-false negative result from balanced ischemia. Hakeem et al. showed patients with CKD with normal MPS still had a three times higher cardiac death rate than those with normal MPS and no CKD (31). In addition, concurrent reduced CFR and LVH may play a role. Fukushima et al. reported CKD patients with normal clinical myocardial perfusion by PET scan had reduced global myocardial flow reserve, which implied an underlying microvascular dysfunction in this population (32) that could explain the poorer prognosis. Increased baseline myocardial blood flow and peripheral endothelial dysfunction in CKD patients have been suggested by Koivuviita et al. (33).

Nonetheless, abnormal MPS results in CKD patients have been shown to be associated with a higher incidence of cardiac events and mortality (34–40). A meta analysis of 12 studies of pre-renal transplant patients showed that the presence of reversible defects of inducible myocardial ischemia was associated with sixfold increased risk of myocardial infarction and almost fourfold risk of cardiac death (41). The presence of fixed defects was associated with a nearly fivefold increased risk of cardiac death (41). Joki et al. suggested that myocardial perfusion abnormalities significantly predicted cardiac events in CKD patients independently of eGFR and left ventricular ejection fraction (34). Among 2967 patients with CKD, the incidence of major adverse cardiac events at 1 year was 1.0, 3.9, 5.9, and 7.3% for normal, mild, moderate, and severe summed stress score, respectively (36). Al-Mallah et al. demonstrated that an interaction between renal function and the magnitude of perfusion deficit assessed by stress MPS in patients with moderate and severe CKD in the presence of abnormal MPS (38).

Blunted heart rate response in CKD patients during stress myocardial perfusion imaging has been reported to be associated with increased mortality (42–44).

### Cardiovascular magnetic resonance

Cardiovascular magnetic resonance (CMR) with gadolinium contrast has not been widely utilized clinically in the CKD population due to the concern of nephrogenic systemic fibrosis (NSF) (45–47). The use of Gadolinium chelates is prohibitive in CKD patients due to the rare but serious side effect of NSF. NSF manifests as a hardening of the skin and internal organs resembling scleroderma, which is irreversible and potentially fatal.

Cardiovascular magnetic resonance spectroscopy has been studied to assess early cardiac dysfunction in pediatric population with advanced CKD (48). Dobutamine stress CMR was shown to be safe in the pre-renal transplant population (49).

## Recommendation

Exercise tolerance has utmost importance both for prognosis and symptoms capacity and has implication in the case of peri-operative risk assessment (50), in this case, renal transplantation. Therefore, we recommend ESE to the case vignette described, to exclude inducible myocardial ischemia, inducible arrhythmia, and to assess exercise capacity and symptoms objectively. Resting echocardiography is useful to exclude significant aortic stenosis prior to exercise stress and to visually assess the degree of LVH.

## Future Developments

Further research is needed for better diagnostic testing for myocardial ischemia in CKD. A new non-contrast blood oxygen level dependent (BOLD) CMR technique has been utilized in several human studies to assess myocardial oxygenation as a measure of ischemia with promising benefits (51–57), namely, in syndrome X, hypertensive patients, patients with CAD, hypertrophic cardiomyopathy, and aortic stenosis. It detects both epicardial and microvascular CAD without the use of potentially toxic Gadolinium chelate contrast agents. The combination of non-contrast BOLD CMR and non-contrast whole-heart magnetic resonance coronary angiography, which can characterize significant proximal epicardial CAD, has potential for assessing myocardial ischemia in the advanced CKD population. This needs to be tested in prospective studies.

## Conflict of Interest Statement

The authors declare that the research was conducted in the absence of any commercial or financial relationships that could be construed as a potential conflict of interest.
